# Homology and Evolution of the Chaetae in Echiura (Annelida)

**DOI:** 10.1371/journal.pone.0120002

**Published:** 2015-03-03

**Authors:** Ekin Tilic, Janina Lehrke, Thomas Bartolomaeus

**Affiliations:** Institute of Evolutionary Biology and Animal Ecology, Rheinische Friedrich-Wilhelms-Universität Bonn, Bonn, Germany; Australian Museum, AUSTRALIA

## Abstract

Echiura is traditionally regarded as a small phylum of unsegmented spiralian worms. Molecular analyses, however, provide unquestionable evidence that Echiura are derived annelids that lost segmentation. Like annelids, echiurans possess chaetae, a single ventral pair in all species and one or two additional caudal hemi-circles of chaetae in two subgroups, but their evolutionary origin and affiliation to annelid chaetae are unresolved. Since annelids possess segmental pairs of dorsal (notopodial) and ventral (neuropodial) chaetae that are arranged in a row, the ventral chaetae in Echiura either represent a single or a paired neuropodial group of chaetae, while the caudal circle may represent fused rows of chaetae. In annelids, chaetogenesis is generally restricted to the ventral part of the notopodial chaetal sac and to the dorsal part of the neuropodial chaetal sac. We used the exact position of the chaetal formation site in the echiuran species, *Thalassema thalassemum* (Pallas, 1766) and *Echiurus echiurus* (Pallas, 1767), to test different hypotheses of the evolution of echiurid chaetae. As in annelids, a single chaetoblast is responsible for chaetogenesis in both species. Each chaeta of the ventral pair arises from its own chaetal sac and possesses a lateral formation site, evidencing that the pair of ventral chaetae in Echiura is homologous to a pair of neuropodia that fused on the ventral side, while the notopodia were reduced. Both caudal hemi-circles of chaetae in *Echiurus echiurus* are composed of several individual chaetal sacs, each with its own formative site. This finding argues against a homology of these hemi-circles of chaetae and annelids’ rows of chaetae and leads to the hypothesis that the caudal chaetal rings evolved once within the Echiura by multiplication of ventral chaetae.

## Introduction

Echiura, consisting of 165 exclusively marine species, is a small, but worldwide distributed taxon of unsegmented spiralians [1–4]. Commonly known as “spoon worms”, due to their tongue-like extensible proboscis, they occur in benthic habitats and range from the littoral zone to the deep sea [5]. Traditionally Echiura was ranked as a phylum, but recent studies, especially molecular, have generated an increasing body of evidence that they actually are derived annelids [6–20] and mostly provide strong support for a sister group relationship between Echiura and Capitellidae. Loss of segmentation in the echiuran stem lineage was substantiated by studies showing serially repeated groups of neurons in the larval nervous system of certain echiuran species [6–8] as well as three subsequently formed pairs of nephridia [21–23] and confirmed with the recent molecular phylogenies [6–20].

**Fig 1:**
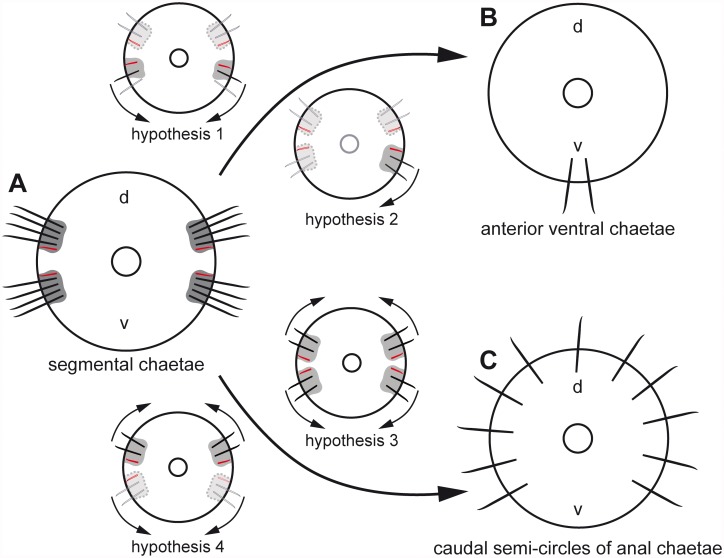
Different hypotheses on the evolutionary transformation of annelid and capitellid segmental chaetae (A) into echiuran ventral (B) and caudal anal chaetae (C). Annelid chaetae are formed on the ventral edge of the notopodial chaetal sac and on the dorsal edge of the neuropodial chaetal sac. Developing chaetae are *red*, completed chaetae are *black*, chaetal sac is *grey*. All structures that are reduced according to the different hypothesis are *paler*; presumably reduced chaetal sacs are marked by a *dotted line*. *Arrows* mark expansion or shifting of chaetal sac. *H1* hypothesis one: notopodial chaetae have been reduced and the neuropodial chaetal sac shifted ventrally. *H2* hypothesis two: notopodial chaetae plus one neuropodial group of chaetae have been reduced and the remaining neuropodial sac shifted ventrally. *H3* hypothesis three: all chaetal sacs expand dorsally and ventrally, respectively. *H4* hypothesis four: neuropodia are reduced and the notopodial chaetae expand dorsally and ventrally.

Like annelids, echiuran species possess chitinous chaetae that arise from a chaetal sac, an ectodermal invagination that generally contains several chaetal follicles and is surrounded by the subepidermal extracellular matrix (ECM). In both annelids and echiurans, each chaeta is formed within one chaetal follicle which consists of a basally located chaetoblast and several follicle cells [24–27]. All cells are epithelial, are interconnected by apical adherens junctions (“belt desmosomes”), face the chaeta and rest on the subepidermal ECM. In annelids, the follicle cells of neighboring follicles are not separated by an ECM and directly contact each other [27–30]. Despite their structural identity, the positional homology of annelid and echiurid chaetae as well as their evolutionary origin is still unsolved. Annelids possess segmental pairs of dorsal (notopodial) and ventral (neuropodial) chaetal sacs, each giving rise to a single row of chaetae. All echiuran species possess a single pair of ventral chaetae. Species of the Urechidae and Echiurinae additionally possess one or two caudal hemi-circles of chaetae, generally called anal chaetae due to their perianal position. Studies of annelid chaetogenesis have revealed that in this group formation of new chaetae is generally restricted to the ventral part of the notopodial chaetal sac and to the dorsal part of the neuropodial chaetal sac, so that neuro-and notopodial formation sites are adjacent on either body side of each segment [27,29]. This pattern is conserved in all species of the Capitellidae, the presumed sister group of the Echiura [11,12,14]. These results allow different sets of hypotheses on the evolutionary origin of the echiuran chaetae (Fig. 1).

Origin of the ventral chaetae: (1) The ventral pair of chaetae in Echiura is homologous to a pair of annelid neuropodia. This hypothesis would be supported, if each ventral chaeta arose from its own chaetal sac and if each should possess a lateral formative site. (2) The ventral pair of chaetae in Echiura is homologous to one neuropodium, while the other one is reduced. This hypothesis would be supported, if both ventral chaetae shared a single chaetal sac with a single formative site.

Origin of the caudal hemi-circles of anal chaetae in Echiurinae and Urechidae: (3) The caudal hemi-circle is homologous to dorsally expanded rows of neuropodial and notopodial chaetae of the annelid ancestor. This hypothesis would be supported, if four chaetal sacs, each with a formative site constituted each hemi-circle of anal chaetae. (4) The caudal hemi-circle is homologous to dorsally merged notopodia of the annelid ancestor; the neuropodia are reduced. This hypothesis would be supported, if only one chaetal sac and two formative sites were found in each caudal hemi-circle of anal chaetae. Any other number of formative sites would falsify both hypotheses and corroborate the results of recent molecular phylogenetic studies. Goto et al. [31] provide convincing evidence for a sister group relationship between Urechidae and Echiurinae and for their placement within the Echiura. Such a position implies that the anal chaetae evolved within the Echiura and leads to the expectation of differences in their mode of formation compared to annelid chaetogenesis.

To test these hypotheses we studied the structure and formation of the ventral chaetae in *Thalassema thalassemum* (Pallas, 1766) and *Echiurus echiurus* (Pallas, 1767) and both hemi-circles of anal chaeta in *E*. *echiurus* using different histological, ultrastructural and immunohistological methods and 3D reconstructions.

## Material and Methods

### Animals

*Thalassema thalassemum* (Pallas, 1766) (Thalassematidae) was collected from rock crevices in the upper sublittoral at Le Cabellou (Concarneau, Brittany, France) in 2007 and 2013, and kept in sea water tanks until fixation. *Echiurus echiurus* (Pallas, 1767) (Echiurinae) was dredged from the Dogger Bank (North Sea) in November 1992 and from muddy sediments in the German Bight (North Sea) (54°02’N, 008°03’E). The animals were kept in sea water tanks up to three years until fixation. *Thallasema thallasemum* specimens were relaxed using a solution of 7% MgCl and seawater (1:1) for 1 hour. The ventral chaetae were then dissected out of the relaxed animals and subsequently fixed for confocal laser scanning microscopy (CLSM) and transmission electron microscopy (TEM). For TEM studies *Echiurus echiurus* specimens were dissected in the fixative. Prior to fixation for histology the specimens were relaxed in a solution of 7% MgCl and seawater (1:1) for 1 hour. In this study six *Echiurus echiurus* specimens were used for preparation, histology and electron-microscopy and twelve *Thalassema thalassemum* specimens were examined histologically, electron-microscopically and by immunostaining.

We neither used endangered species nor were the investigated animals collected in a protected area. All animals were collected with the permission of the local marine biological stations. The Station Biologie Marine, Concarneau, was informed of our collection of *Thallasema thalassemum*. No written permission was necessary. *Echiurus echiurus* was collected subtidally using the research vessel "Uthörn" of the Alfred-Wegener-Institute for Polar and Marine Research. No written permission to collect the animals was necessary.

### Transmission electron microscopy (TEM)

For electron microscopy ventral chaetal sacs of *Thalassema thalassemum* were fixed in 2.5% glutaraldehyde buffered in 0.05 M phosphate buffer with 0.3 M NaCl for 1hour. *Echiurus echiurus* was fixed in 2.5% glutaraldehyde buffered in 0.1 M sodium cacodylate (pH 7.2) at 4°C for 90 min. After having rinsed the specimen several times in the same buffer used for fixation, they were postfixed in 1% OsO_4_ buffered in 0.05 M phosphate 0.3 M NaCl saline or in 1% OsO_4_ buffered in 0.1 M sodium cacodylate buffer, respectively. All specimens were dehydrated in an ascending acetone series and propylene oxide and subsequently embedded in Araldite. The basal part of a chaetal follicle and the formative sites were sectioned into a complete series of 70 nm silver-interference coloured ultra-thin sections, stained with uranyl acetate and lead citrate using an automated TEM stainer (QG-3100) and analyzed in a ZEISS Libra-120KV transmission electron microscope.

### Confocal laser scanning microscopy (CLSM)

Ventral chaetae of *Thalassema thalssemum* were fixed in 4% paraformaldehyde and subsequently permeabilized in four 5-min changes of PBS (phosphate buffered with saline) with 0.1% Triton X-100 (Fisher Scientific). The preparations were then stained overnight in 4°C with TRITC phalloidin at a dilution of 1:100 (in 0.3% Triton X-100). After staining, the samples were rinsed in three quick changes and subsequently in two 10-min changes of PBS with 0.1% Triton and one 10-min rinse in PBS without Triton. The chaetae were then directly placed in hollow-ground slides. The samples were quickly dehydrated in isopropanol (2 min 70%, 2 min 85%, 2 min 95%, 2 min 100%, 2 min 100%), cleared in three 15-min changes of Murray Clear, mounted in Murray Clear, and sealed with nail polish.

For the propidium iodide induced staining of the nuclei, paraformaldehyde fixed ventral chaetae of *T*. *thalassemum* were treated with RNase (1: 100) for 30 min and subsequently stained with propidium iodide (1:40) for another 30 mins. The samples were cleared in an ascending series of glycerol in PBS (15 min 30%, 30 min 60%, 30 min 90%) and mounted in 90% glycerol.

### Histology and 3D reconstruction

For semi-thin sections, Araldite-embedded ventral chaetal sacs of *Thalassema thalassmum* were cut into complete series of 0.5 μm sections with a diamond knife (Diatome Histo Jumbo) on a Leica Ultracut S ultramicrotome [32]. The sections were stained with toluidine blue (1% toluidine, 1% sodium-tetraborate and 20% saccharose) and mounted with araldite. The sections were then analyzed with an Olympus microscope (BX-51) and photographed with an Olympus camera (Olympus cc12) equipped with the dotSlide virtual slide system (Olympus).

For paraffin histology *Echiurus echiurus* specimens were fixed in Bouin’s fixative for 18 h in room temperature and transferred into 70% ethanol. Herein, the animals were sectioned to isolate the caudal section with the hemi-circles of anal chaetae, and the anterior part containing the ventral pair of chaetae. Prior to embedding one specimen was photographed with a Keyence VHX700 digital microscope. The different parts of *Echiurus echiurus* were then completely dehydrated in an ethanol series, followed by incubation in methyl benzoate and butanol. Afterwards each part was pre-incubated in Histoplast (Thermo Scientific, Dreieich, Germany) at 60°C for three days with several medium changes and finally embedded in Paraplast (McCormick Scientific, Richmond, USA). 10 μm thick sections were made using a Reichert-Jung Autocut 2050 microtome (Leica, Wetzlar) and transferred to glass slides coated with albumen-glycerin. Sections were AZAN stained; Malinol (Waldeck, Münster, Germany) was used for mounting the sections.

The histological sections were analyzed with an Olympus microscope (BX-51) and photographed with an Olympus camera (Olympus cc12). The images of the posterior part of *Echiurus echiurus* were aligned with IMOD (Boulder laboratories, [33]) and IMOD-align (http://www.evolution.uni-bonn.de/mitarbeiter/bquast/software). Selected histological images were used as reference for the 3D model generated with the software 3ds max 13.0. The histological images were imported as surface materials and the chaetae were modeled using standard cylindrical or conic objects. These were modified as NURBS-surfaces.

### Voucher material and data repository

A conspecific individual sampled form the same field site as the studied animals has been sequenced. The partial 16S sequence has been deposited in NCBI and is available under the accession number GenBank: KM187648.1. Aligned serial semi-thin sections of the ventral chaetae of *Thalassema thalassemum* are freely accessible in the morphological database, MorphDBase [34]. Direct link: https://www.morphdbase.de/?E_Tilic_20140929-M-21.1

## Results

### Ventral chaetae in *Thalassema thalassemum* and *Echiurus echiurus*

**Fig 2:**
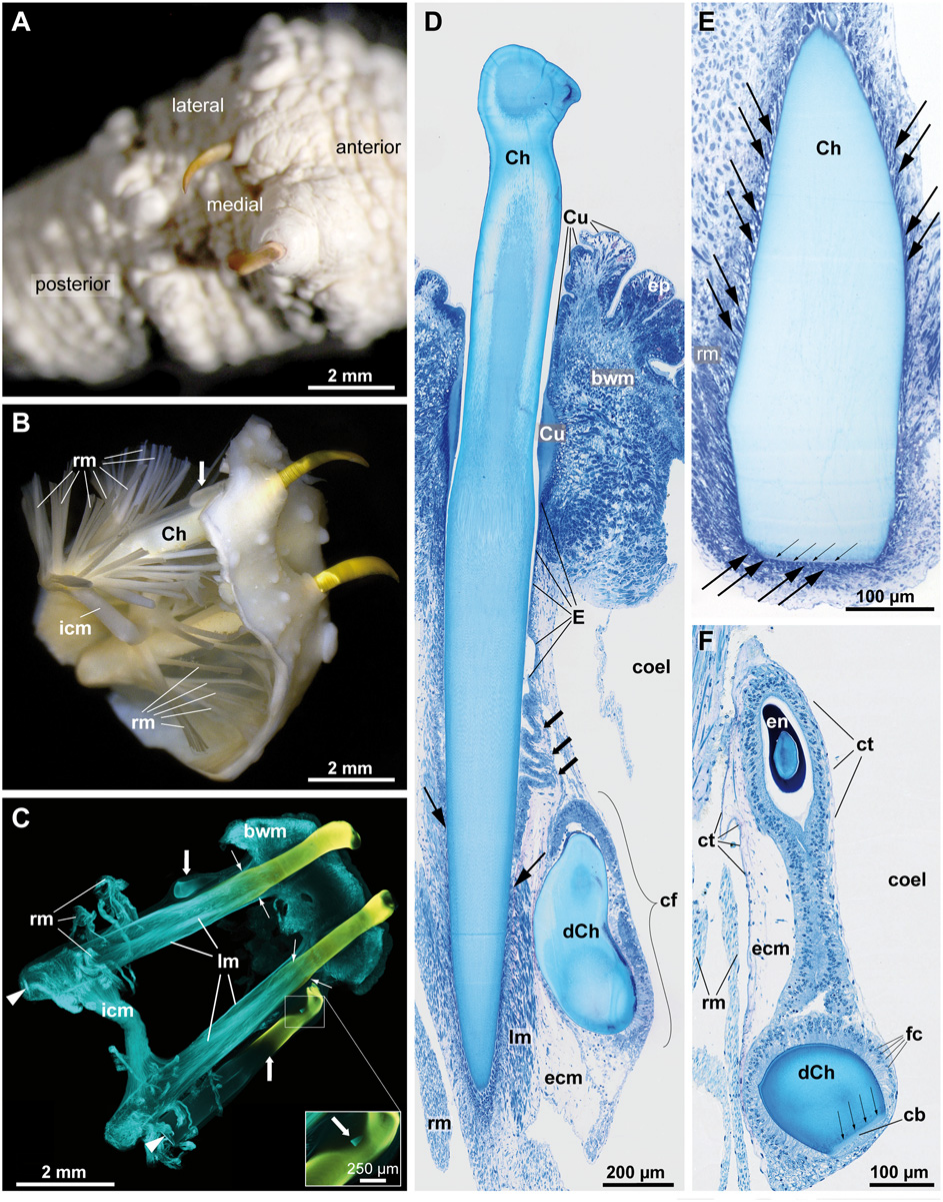
Ventral chaetae of *Echiurus echiurus* (A, B) and *Thalassema thalassemum* (C-F). A. Extended focus light micrograph of a critical point dried part of a specimen showing the apical hooks of the ventral chaetae. B. Extended focus light micrograph, frontal view of a pair of dissected ventral chaetae (*Ch*) showing the chaetal radial muscles (*rm*) and interconnecting muscle (*icm*). Chaetal sacs are delicate and transparent, *arrow* marks a developing chaeta. C. Maximum projection of a phalloidin (*cyan*) stained and chitin autofluorescent (*yellow* to *light blue*) dissected pair of ventral chaetae, frontal view, CLSM. Apical microvilli of the chaetoblast marked by *arrow head*s. *Large arrows* mark developing chaetae, *small arrows* mark the collar of the ventral chaetae. Inset: early chaetogenesis at higher magnification. D.-F. Light micrographs of semi-thin (0.5 μm) toluidine blue stained parasagittal sections. D. Apical part of ventral chaeta (*Ch*), chaetal sac with follicles (*bold arrows*, *cf*) and developing chaeta (*dCh*). Large arrows mark the uppermost follicle cells. Note muscles running between follicles. E. Basal part of a completed ventral chaeta, *large arrows* mark follicle cells with their strong bundles of intermediate filaments, *small arrows* mark microvilli brush border of chaetoblast. F. Developing chaeta (*dCh*) with chaetoblast (*cb*) plus microvilli brush border (*small arrows*) and follicle cells (*fc*). Note that intermediate filaments are absent at this stage. Coelothel (*ct*) surrounds the follicle except for the apical section. *bwm* body wall muscles, *coel* coelom, *Cu* cuticle, *ep* epidermis, *ecm* extracellular matrix, *en* enamel, *icm* interconnecting muscle, *lm* longitudinal muscle, *rm* radial muscle.

In all specimens studied two large golden chaetae extend from the ventral surface, one on either side of the ventral midline of the body. The tip of the chaeta is curved and bent towards the animal’s surface. In relaxed animals only the curved, hook-like section of the chaeta is externally visible (Fig. 2A-B). Underneath this hook-like section the diameter of the chaeta initially decreases slightly, then increases rapidly to form a collar and is finally more or less constant until the base of the chaeta (Fig. 2C-D). Below, the collar will be used as a landmark for describing the follicle. Depending on the age and the size of the animal, the ventral chaetae of *Thalassema thalassemum* are up to 3 mm long, those of *Echiurus echiurus* are up to 9 mm long; in any case the length of both chaetae is almost identical.

### Chaetal sac and chaetal follicles

**Fig 3:**
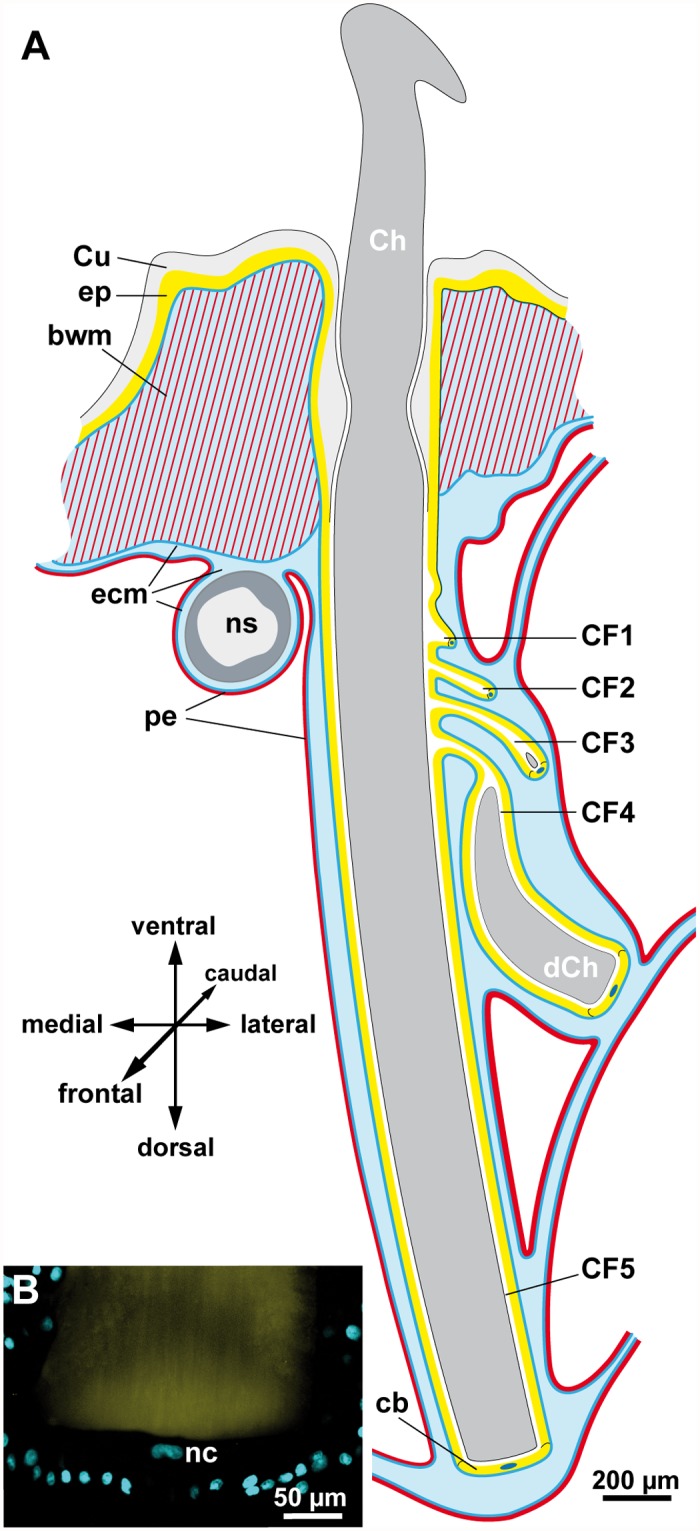
Chaeta and chaetogenesis in *Thalassema thalassemum* reconstructed from a stack of semi-thin sections (Direct link: www.morphdbase.de/?E_Tilic_20140929-M-21.1). A. Chaetal sac with four developing chaetal follicles (*CF1*–*CF4*) and one completed follicle (*CF5*). Developing chaetae (*dCH*) in *CF3* and *CF4* and complete chaeta (*Ch*) of CF5 are *dark grey*. Chaetal muscles are not shown. B. Maximum projection of a CLSM stack of the basal section of CF5 with propidium iodide staining of nuclei (*cyan*) and autofluorescent chaeta (*yellow*). Note large nucleus (*nc*) of chaetoblast. *bwm* body wall muscles, *cb* chaetoblast, *Cu* cuticle, *ecm* extracellular matrix, *ep* epidermis, *ns* ventral nerve cord, *pe* peritoneum.

Each chaeta arises from one chaetal sac that deeply invaginates into the trunk coelom. The chaetal sac is composed of a large, main follicle and a few developing ones and voluminous perifollicular ECM with muscles and is surrounded by a sheath formed by the peritoneum (Figs. 2D-F and 3A). Muscle bundles originate from the basal section of the chaetal sac and cross the coelomic cavity. A large interconnecting muscle links the chaetal sac of one body side to its counterpart on the other body side and several radial muscles connect the chaetal sac to the body wall (Fig. 2B). All muscles are at least partly surrounded by a peritoneum and consist of fiber muscle cells. In addition several smaller muscles run within the chaetal sac, most of them parallel to the main chaeta (Fig. 2C-E). The course and orientation of the muscles are similar in both species studied.

In both species studied the largest and fully differentiated chaeta originates from the main, largest follicle of each chaetal sac (Figs. 2D and 3A). Along its course from apical to basal, its inner lining initially is continuous with the epidermis and thus cuticularized. This cuticle can be seen to the level of the chaetal collar, since it drastically increases in diameter directly above the chaetal collar, decreases there and finally disappears (Fig. 2D). Here, the main chaetal follicle forms a series of lateral pouches that increase in size along an apico-basal gradient. The pouches are developing chaetal follicles. The last and most basally located pouch always contains a developing chaeta (Figs. 2D, F and 3A). Depending on the size and age of this chaeta, a further developing chaeta may be present in the adjacent follicular pouch (Figs. 2C and 3A). In all specimens studied, the size of the developing chaetae differed between body sides, indicating that chaetogenesis is not initiated at the same time within the two separate chaetal sacs of an individual.

Each follicle consists of several hundred follicle cells and a basal chaetoblast; all cells are epithelial and rest on the perifollicular matrix (Figs. 2E and 3A). The size of the nuclei differs between follicle cells and chaetoblast in both species. While the former are ovoid in shape and measure 7.2 ± 0.8 μm (n = 24) in length and 4 ± 0.6 μm (n = 20) in diameter, the nucleus of the chaetoblast of fully differentiated chaeta is disc-shaped with a central depression and measures 21–23 μm in width and 8–8.5 μm in diameter (Fig. 3B). The metrical data on the nuclei do not differ significantly between both species.

**Fig 4:**
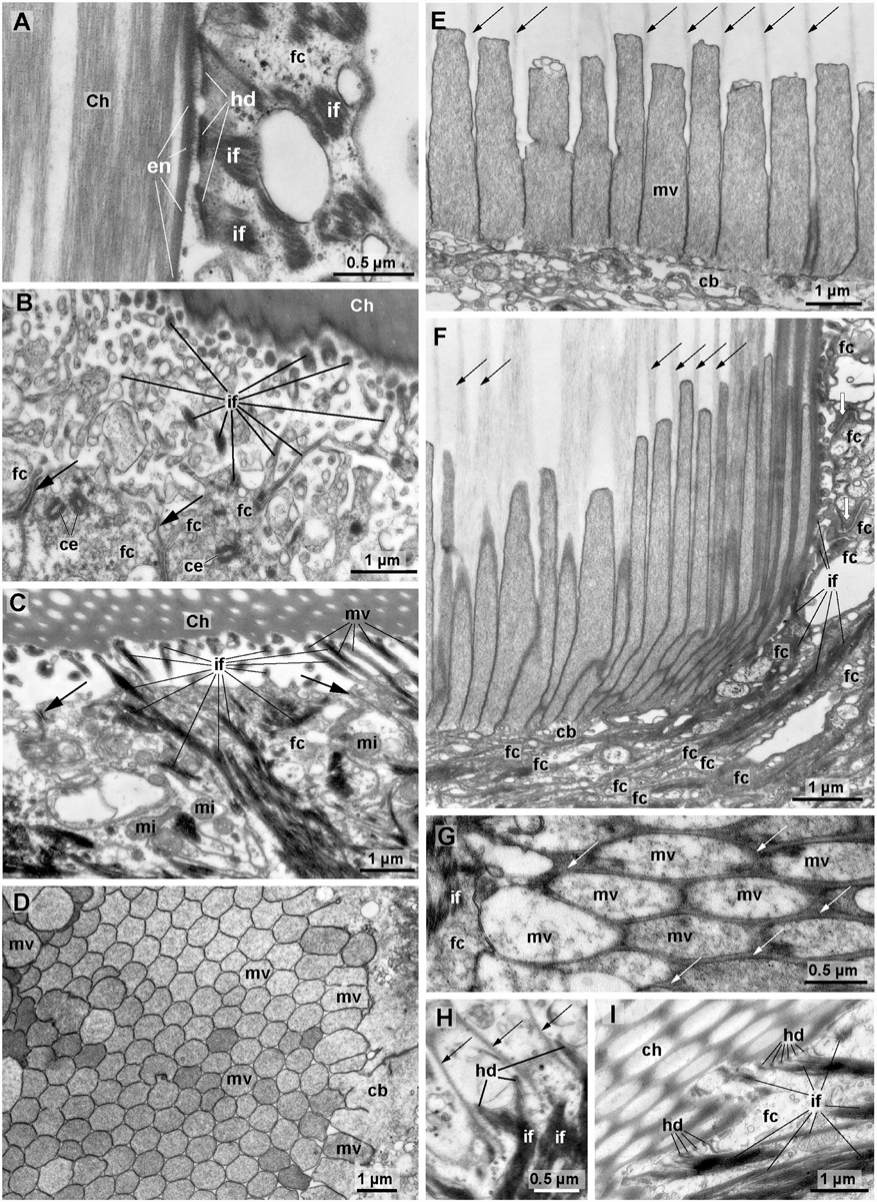
Ultrastructure of ventral chaetae of *Echiurus echiurus* (A, D-F) and *Thalassema thalassemum* (B, C, G-I), TEM. A-G. late chaetogenesis, H-I. complete chaeta. A. Hemidesmosomes (*hd*) connect chaeta (*Ch*) to intermediate filaments (*if*) of follicle cells (*fc*), chaeta is surrounded by an enamel (*en*). B, C. Intermediate filaments (*if*) inside branched and unbranched microvilli (*mv*) adhere chaeta to follicle cells (*fc*). Apical adherens junctions (*arrows*) interconnect follicle cells, note apical pair of centrioles (*ce*) in follicle cells in B. D. Semi-transverse section of microvilli (*mv*) brush border of a chaetoblast (*cb*) during chaetogenesis. E, F. Parasagittal section of chaetoblast with apical microvilli, *arrows* mark chitin polymerizing between the microvilli. Note densely packed f-actin in microvilli (*mv*). E. center of chaetoblast. F. lateral part of chaetoblast. Note its tight interdigitation with follicle cells (*fc*), *white arrows* mark adherens junctions. G. Semi-transverse section of apical microvilli of the chaetoblast, *white arrows* mark chitin polymerizing between the microvilli (*mv*). H. Base of old chaeta, parasagittal section. The apical microvilli have been replaced by protrusions of the chaetoblast that contain intermediate filaments (*if*) which adhere to the chaeta (*arrows*) by hemidesmosomes (*hd*). I. Fully differented chaeta with ridges. Hemidesmosomes (*hd*) firmly connect intermediate filaments (*if*) of follicle cells (*fc*) to chaeta. *mi* mitochondria.

Follicle cells of both species contain strong bundles of intermediate filaments (Fig. 4A-C, F, I). Apically, the intermediate filaments extend into branched or unbranched protrusions of the cell surface (Fig. 4B, C, I). In the tip of these protrusions the intermediate filaments adhere to hemidesmosomes that connect them to the chaeta (Fig. 4A, C, I). In their basal section ventral chaetae of *T*. *thalassemum* contain shallow ridges so that the outer chaetal surface to which the follicle cells adhere is much larger than in *E*. *echiurus* which lacks such ridges (Fig. 4A, I). Hemidesmosomes on the basal surface of the follicle cell connect the intermediate filaments to the ECM. Opposite to the hemidesmosomes dense plaques of fiber muscle cells adhere to the ECM, so that the follicular intermediate filament system mechanically couples the muscles to the chaeta (Fig. 2E).

The chaetoblast is also filled by strong bundles of intermediate filaments that reach into short apical protrusions that extend into the inner of the chitinous tubes of which the chaeta is composed (Fig. 4H). Here, hemidesmosmes connect the intermediate filaments of the chaetoblast to the chaeta. The chaetoblast of differentiated chaetae forms a very small cytoplasmic layer underneath the chaeta, contains large amounts of intermediate filaments, a few microvilli and vesicles and the large nucleus (Fig. 3B). The chaetoblast is flanked by follicle cells and intensely interdigitates with these, so that the chaetoblast can hardly be discriminated from the latter without using electron microscopy.

### Chaetogenesis

Chaetogenesis is a continuous process in both echiurid species during which the number of follicle cells as well as the size of the chaetoblast and its nucleus increases (Fig. 3A). Lateral to the main follicle a series of young follicles increasing in size and age can be seen (Figs. 2D and 3A). The follicle next to the main follicle always contains a developing chaeta (Figs. 2C-D and 3A). Each consists of a large number of follicle cells and a basal chaetoblast (Fig. 2F). As in fully differentiated chaetae follicle cells are joined to the developing chaetae by hemidesmosomes that connect the chaeta to the intercellular intermediate filament system. In *T*. *thalassemum* these hemidesmosomes are located at the tip of branched apical protrusions, which may be several micrometers long (Fig. 4B-C). The follicle cells always contain a subapical pair of centrioles; basal structures, rootlets or any other sign of ciliogenesis are missing (Fig. 4B). We therefore assume that the centrioles indicate ongoing mitotic activity of the follicle cells, which is needed to increase the number of follicles during growth of the chaeta. The chaetoblast can easily be recognized by its stout apical microvilli that measure 3.83 ± 0.29 μm (n = 15) in length in *E*. *echiurus* and 6.48 ± 0.52 μm (n = 10) in *T*. *thalassemum* (Fig. 4D-F). Although shorter, the microvilli of the chaetoblast in *E*. *echiurus* are larger (740 ± 75 nm in diameter (n = 28)) than those in *T*. *thalassemum* (425 nm ± 63 nm in diameter (n = 28)). The size of the microvilli is identical throughout each chaetoblast except for the periphery of the chaetoblast, where it decreases in both species (Fig. 4D). The microvilli are densely filled with actin filaments that extend into the chaetoblast where they interconnect and form a subapical meshwork (Fig. 4D-E). During chaetogenesis fibrillar electron-grey material, which presumably is chitin, is deposited between the microvilli and elongates the chaeta (Fig. 4E-F). Since the length of the microvilli is more or less fixed, empty tubes remain that the microvilli once filled. These tubes can be seen in any cross section of the chaeta, forming the honey-comb pattern (Fig. 4I)

During the further course of chaetogenesis the chaetae are coated by an enamel, which is very thick in the apical section of the chaeta and absent in the basal section (Fig. 2F). The source of the material forming this enamel was not seen. We assume that it is released by the follicle cells, because these are the only ones that are next to the apical part of the chaeta. During early chaetogenesis the follicle cells contain no intermediate filaments (Fig. 2F). These are formed later during chaetogenesis and increase in number, until they finally dominate the follicle cells and can even be seen at the light microscopical level (Fig. 2D-E). Intermediate filaments finally drastically reduce the actin filaments which had been the prevailing filament system during chaetogenesis and restrict it to the periphery of the chaetoblast. Short apical protrusions of the chaetoblast connect the chaeta to the chaetoblast. These processes contain intermediate filaments that adhere to the chaeta by hemidesomosmes (Figs. 2C and 4H). Whether the microvilli that once preformed the chaeta are transformed into these processes or are replaced by them remains to be shown. When completed the chaeta replaces the old, used chaeta, which seems to be cast off. Shortly before this the old follicle changes its structure. There are lesser intermediate filaments in the follicle cells, their diameter decreases and the stainability with Azan staining changes. Ultrastructural details of these processes and the loss of used chaetae were not observed.

### Anal chaetae in *Echiurus echiurus*

**Fig 5:**
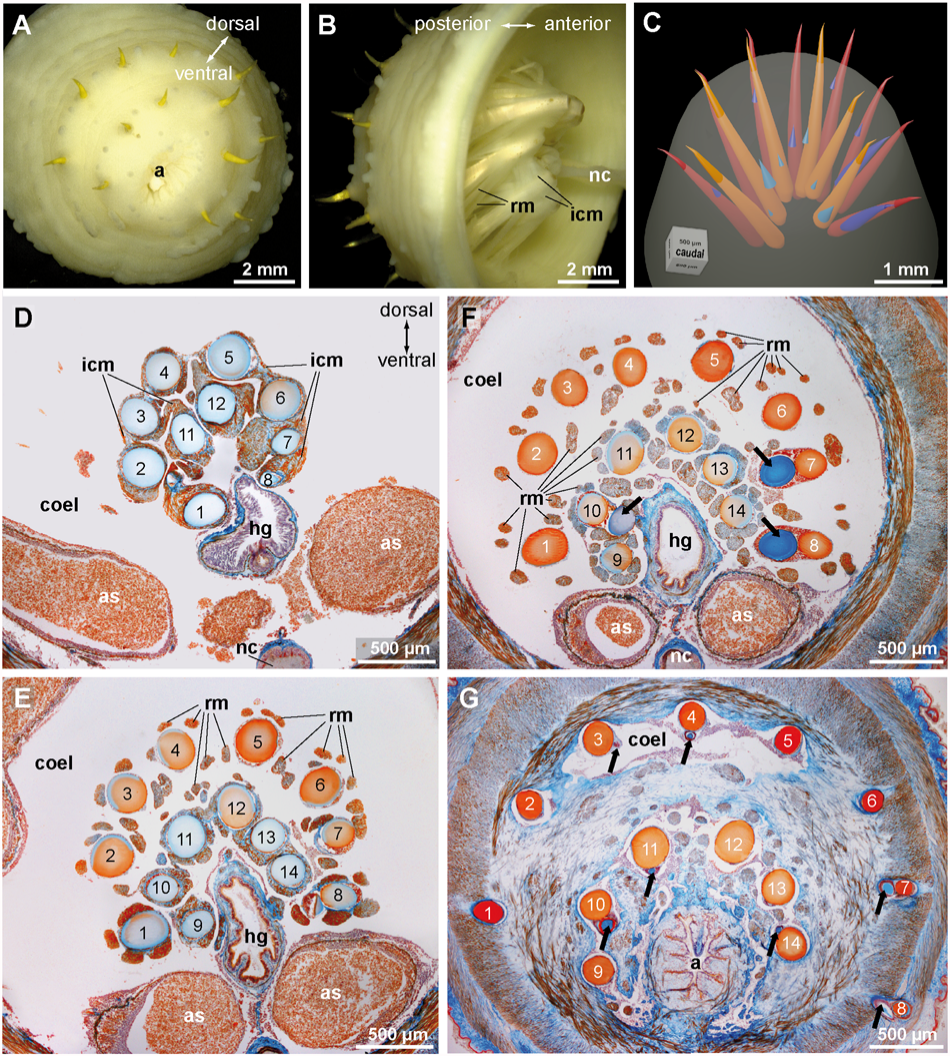
*Echiurus echiurus*, anal chaetae. A. Extended focus view of the caudal face of the animal, B. Extended focus semi-lateral view into the dissected caudal end; Hindgut is removed, nerve cord (*nc*) marks ventral side. Note complex muscular system, interwoven interconnecting muscles (*icm*), and twisted orientation of anal chaetae. C. Reconstruction of completed (*orange*) and developing (*bluish*) anal chaetae based on a series, D-G. Azan stained histological sections from a series of transverse sections from anterior to posterior. The chaetal sacs extend deeply into the coelom (*coel*), the anal chaeta are numbered (outer hemi-circle 1–8, inner hemi-circle 9–14). Distances: D-E = 600 μm, E-F = 200μm, F-G = 600 μm. D. Section of the base of the anal chaetae dorsal to hindgut. Note connection of chaetal sacs by interconnecting muscles (*icm*). E. Section of the chaetae at the level of the origin of the radial muscles (*rm*). Anal chaetae are grouped in two hemi-circles that are next to each other and partly surround the hindgut (*hg*). F. Section close to the posterior end of the trunk body cavity (*coel*). Hemi-circles of anal chaetae are clearly separated and surround three quarters of the hindgut. Note developing chaeta (*arrow*) in the sacs of chaetae 7, 8, and 10. G. Section of the anal chaeta through the body wall muscles. Hemi-circles of anal chaetae are widely separated. Note tips of developing chaetae (*arrow*) in sacs of chaetae 3, 4, 7, 8, 10, and 11. *a* anus, *as* anal sac.

*Echiurus echiurus* possesses anal chaetae that are arranged in two caudal, horse-shoe shaped to hemi-circular rows that surround the anus dorsally; chaetae are absent caudo-ventrally (Fig. 5A). Each chaeta is slightly curved with a bent tip that extends beyond the surface of the animal, while the basal section of each chaeta extends deeply into the body cavity of the animals (Fig. 5B). In the animal studied histologically, the anal chaetae measure 2.7 ± 0.5 mm (n = 14) in length and 277 ± 17 μm (n = 20) in diameter at its base (Fig. 5B-C). Depending on the size and age of the individual, the anal chaetae may be larger or smaller, but always have more or less the same size in one individual. The outer hemi-circle consists of 7 to 8 chaetae, the inner of 6 chaetae (Fig. 5A-C). The numbers corroborate the data in Spengel [24].

Each chaeta arises from one chaetal sac which is surrounded by extracellular matrix. Muscles rest on the coelomic face of the matrix, while follicle cells rest on its opposite side and directly face the chaeta. The basal section of each chaetal sac is connected to its neighbors by interconnecting muscles the myofilaments of which are perpendicular to the main axis of the chaetae (Fig. 5D). Towards the tip of the chaetae these interconnecting muscles are replaced by radial muscles that originate from the chaetal sac and cross the coelom and adhere to the body wall muscles at the caudal margin of the trunk coelom (Fig. 5E-G). There are up to seven of these muscles per chaetae; they seem to form a collar of muscle strands around each of the caudal chaetae (Fig. 5B).

Chaetogenesis could be seen in all chaetal sacs of the specimen studied histologically (Fig. 5F-G). Developing chaetae are each found inside their own chaetal follicle, where they are surrounded by several follicle cells. Within each chaetal sac the developing follicle is adjacent to the chaetal follicle of the fully grown chaeta and not separated from the latter by an ECM. 3D reconstruction revealed that only one developing chaeta is found in each chaetal sac (Fig. 5C). This reconstruction also shows that the developing chaeta differ in length and position within each chaetal sac, a finding which indicates that new chaetae are formed individually within each chaetal sac. All these results clearly show that the anal chaetae of *Echiurus echiurus* independently arise from several chaetal sacs that are arranged as a hemi-circular row surrounding the hindgut.

## Discussion

The ultrastructure of echiuran chaetae is similar to those of Annelida (Echiura: Orrhage [25]; Annelida: see Bartolomaeus[29]; Hausen [27]; Specht and Westheide [28] for review). Fibrillate chitinous material fills the gaps between the tubes which are left by the microvilli of the chaetoblast during chaetogenesis. The chaetoblast is located at the base of an epidermal chaetal follicle and, as long as a chaeta grows, releases chitin into the gap between its microvilli bases that polymerizes to elongate the chaeta. Since the length of the microvilli is more or less fixed, empty tubes remain that the microvilli once filled. These tubes can be seen in any cross section of the chaeta, forming the honey-comb pattern characteristic for annelid chaetae. These tubes may later be filled by some electron-dense material, so that the honey-comb pattern is less clearly visible than in transverse sections of the proximal part of the chaeta.

Like in annelids (O’Clair and Cloney [35] for *Nereis vexillosa*, Bartolomaeus and Meyer [36] for Arenicolidae, Tilic et al. [30] for *Lumbrineris* species) chaetal formation is in accordance with continuous adjustment of the apical microvilli pattern of the chaetoblast in both echiurid species. In both species modulation merely concerns an increasing number of microvilli during chaetogenesis along with a reorientation of the main axis of the chaetoblast. Increasing number of microvilli in time leads to chaetae with a small tip and a broad base, while reorientation of the chaetoblast’s axis causes the hook-like apical section of the ventral chaeta. Such a re-orientation was not seen in the caudal chaetae of *Echiurus echiurus* which lack an arcuate apical section. After the chaeta has been completed the microvilli of the chaetoblast are replaced by protrusions containing intermediate filaments. Such a replacement has also been reported from annelids and marks the end of chaetogenesis [37].

Chaetoblast and follicle cells are epithelial cells. While the chaetoblast determines the structure of the chaeta in annelid and echiruan species, the follicle cells connect it to the muscular system by an intracellular meshwork of intermediate filaments [27]. In addition to these mechanical functions, the follicle cells may produce the enamel that surrounds the chaeta. A very large number of follicle cells lines the chaetal follicle in both echiuran species. It seems likely that their number increases during chaetal formation by mitosis. In both echiuran species developing chaetae are located laterally to the fully formed chaetae within a separate chaetal follicle. The younger the chaetae are, the closer they are located to the animal’s surface. During chaetogenesis the young follicle continuously sinks into deeper tissue layers. The position of the formative site as well as spatial re-orientation of the developing chaeta is identical in annelids [29, 27]. As in annelids, each chaetal sac consists of a few chaetal follicles, but in contrast to annelids only one chaeta of each sac extends beyond the animal’s surface. Each externally visible chaeta thus marks a single chaetal sac.

Presently we have no detailed insight into degeneration of echiuran chaetae, but the fact that chaetae are continuously formed in a chaetal sac provides indirect evidence for replacement of the chaeta. Due to the general course of chaetogenesis we conclude that replacement of chaetae initiates the degeneration of the follicle. In annelids chaetal degeneration has been described in a very few species, although it should be an integral part of each chaetal sac’s developmental history [27,38].

To us, however, the most surprising finding was that a single chaetoblast forms the ventral chaetae which may measure more than half a millimeter in diameter. Given that the entire apical surface of the chaetoblast is densely covered by microvilli that are a few micrometers long, the surface of the chaetoblast must be very large. The requirement for the continuous release of chitin suggests that the physiological activity of this cell must be very high. These considerations might explain the hypertrophied nucleus, which according to the low intensity of the propidium iodide staining, relative to the nuclei of the follicle cells, is despite of its size probably not polyploid (Fig. 3B).

### Comparison and evolutionary significance of chaetae in Echiura

**Fig 6:**
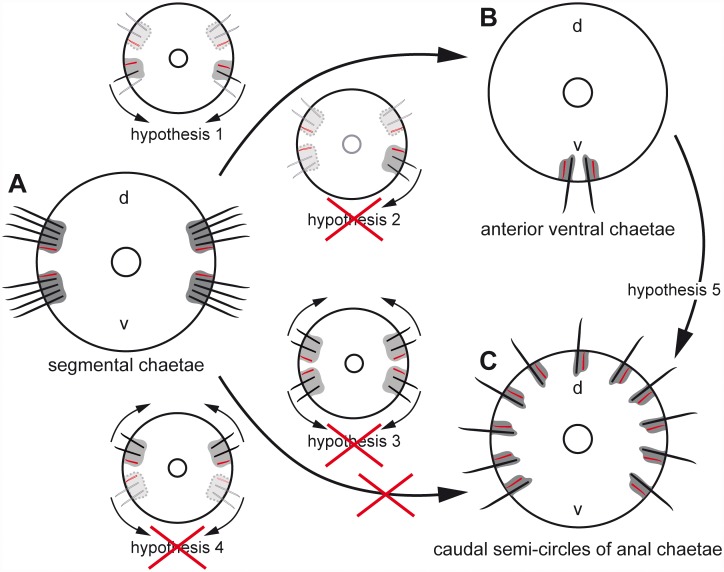
Rejection, support and new hypotheses on the evolutionary transformation of the segmental chaetal pattern characteristic for Capitellidae and most other Annelida (A) into that of Echiura (B, C). The number of the ventral chaetal sacs and position of the growth zone supports the first hypothesis by suggesting that the ventral chaetae evolved from neuropodia by reduction of the number of chaetae and loss of the neuropodia. All other hypotheses are rejected by the results of this paper. A new hypothesis (hypothesis 5) is developed for the evolution of the anal chaetae in Urechidae and Echiurinae, according to which the anal chaetae evolved by multiplication of ventral chaetae.

As expected from the assumed position of Echiura as an ingroup of the Annelida [9,11,14], the chaetae, the composition of chaetal follicles and chaetal sacs, ultrastructure, and chaetogenesis are similar in these two taxa. Presently, however, we are unable to detect support for a specific sister group relationship to any annelid subgroup, especially not for the Capitellidae, which turn out to be the echiuran sister group when phylogenetically analyzing single genes and phylogenomic data [11,12,14]. Capitellid species possess dorsal and ventral pairs of capillary chaetae in the thorax and hooded hooks in the abdomen [39]. Each row originates from a chaetal sac and within each sac the formative site is located close to the lateral mid-line of the animal (Fig. 6). Chaetogenesis thus is restricted to the ventral edge of the notopodial chaetal sac and to the dorsal edge of the neuropodial chaetal sac. Such a position of the formative site within the different chaetal sacs is assumed to be ancestral in Annelida [27,40]. Although our study does not provide morphological evidence for a Capitellidae-Echiura sister group relationship, our initial hypotheses on the evolution of echiruan chaetae (Fig. 1) can be evaluated.

The presence of two separate ventral chaetal sacs, each with a single chaeta extending beyond the animal’s surface and a lateral site of chaetal formation, supports our first hypothesis on the transformation of annelid neuropodial chaetae into echiuran ventral chaetae (Fig. 6). The second hypothesis that predicted a single ventral sac with a single formative site must therefore be rejected. Provided that Echiura and Capitellidae are sister groups, as supported by molecular data [11,12,14], the ventral chaetae of the Echiura must be homologous to the ventral chaetae of Capitellidae. Since the echiuran chaetae do not show any sign of the hood characteristic of the abdominal chaetae of Capitellidae [39], the ventral chaetae should be homologous to thoracic neuropodia, most likely to those of the first chaetiger, because the ventral chaetae in Echiura are the first (and in most species the only) chaetae that are formed during development. We therefore assume that the notopodial chaetae of a common echiurid-annelid ancestor were lost and that the neuropodial chaetae of this segment shifted from a medial to a ventral position (Fig. 6).

The presence in *Echiurus echiurus* of one chaetal sac per anal chaeta, each with its own lateral formative site, argues against the homology of the caudal hemi-circles of chaetae to the neuropodia or notopodia of capitellids or any other annelid species. Provided that urechidan and echiurine anal chaetae are formed in an identical manner, hypotheses 3 and 4 are thus rejected. Recent molecular studies actually provide evidence that Echiurinae and Urechidae are sister taxa within the Echiura [31, 41]. A caudal ring of anal chaetae is found in all urechid species and two hemicircles of such chaetae in all echiurid species [4], such a sister group relationship implies that the specific arrangement of anal chaetae either represent an autapomorphy of the respective group or that one of these arrangements evolved in their common stem lineage and is plesiomorphic. The direction of evolution remains unknown.

If urechid species also have anal chaetal sacs with one chaeta and lateral formation sites, like the ones described here for Echiurus echiurus, we propose that anal chaetae evolved in a common stem lineage of Echiurinae and Urechidae. Since each of these chaetal sacs is identical to the ventral chaetal sacs, we assume that they evolved by multiplication and rearrangement of the same anlagen that give rise to ventral chaetae (Fig. 6). This hypothesis has to be tested by studies of the ontogeny of representatives of Urechidae and Echiurinae. Presently, this hypothesis is mainly supported by the derived position of these sister taxa nested within Echiura [31, 41] and the muscular system that is almost identical in ventral and anal chaetae in consisting of interconnecting and radial muscles.
